# Alcohol-Related Intimate Partner Violence Among White, Black, and Hispanic Couples in the United States

**Published:** 2001

**Authors:** Raul Caetano, John Schafer, Carol B. Cunradi

**Affiliations:** Raul Caetano, M.D., Ph.D., is a professor of epidemiology and assistant dean at the University of Texas, School of Public Health, Houston, Texas. John Schafer, Ph.D., is an associate professor in the Department of Psychology, University of Cincinnati, Cincinnati, Ohio. Carol B. Cunradi, M.P.H., Ph.D., is an associate research scientist at the Prevention Research Center, Berkeley, California

**Keywords:** AODR (alcohol or other drug [AOD] related) violence, domestic violence, spouse abuse, ethnic differences, African American, Hispanic, White American, gender differences, disinhibition theory of AODU (AOD use, abuse, and dependence), predictive factors

## Abstract

Intimate partner violence (IPV) is a major public health problem in the United States. Results from a 1995 national study indicated that 23 percent of the black couples, 11.5 percent of the white couples, and 17 percent of the Hispanic couples surveyed reported an incident of male-to-female partner violence in the 12 months preceding the survey. The rate of female-to-male partner violence was also high: 15 percent among white couples, 30 percent among black couples, and 21 percent among Hispanic couples. The higher prevalence of IPV among ethnic minorities, compared with whites, cannot be explained by any single factor, but seems to be related to risk factors associated with the individual, the type of relationship between partners, and factors in the environment. Alcohol plays an important part in IPV. The study found that 30 to 40 percent of the men and 27 to 34 percent of the women who perpetrated violence against their partners were drinking at the time of the event. Alcohol-related problems were associated with IPV among blacks and whites, but not among Hispanics. Alcohol’s role in partner violence may be explained by people’s expectations that alcohol will have a disinhibitory effect on behavior or by alcohol’s direct physiological disinhibitory effect. It is also possible that people consciously use alcohol as an excuse for their violent behavior or that alcohol appears to be associated with violence because both heavier drinking and violence have common predictors, such as an impulsive personality.

Researchers have studied intimate partner violence (IPV) by listening to victims’ reports and by conducting surveys of the general public that ask participants whether they have perpetrated this type of violence themselves. The acts of violence considered by researchers are varied, ranging from slapping and pushing (i.e., moderate violence) to battery or using guns and knives (i.e., severe violence). Studies conducted among patients in treatment for substance abuse problems, victims of battering in women’s shelters, and people selected from household samples in the community indicate that a high rate of IPV occurs in the United States. For instance, results from the National Family Violence Resurvey of 1985, which examined overall rates of IPV among couples in the general population, indicated that about 17 percent of all U.S. couples experienced at least one episode of IPV sometime in the 12 months prior to the survey (Straus and Gelles 1990). Rates of severe partner violence (e.g., battery, rape, and assault with a knife or gun) are lower. In the 1992 National Alcohol and Family Violence Survey, approximately 2 percent of couples reported a severe husband-to-wife assault, and 4.5 percent of couples reported a severe wife-to-husband assault (Straus and Kantor 1994). The rate for severe wife-to-husband violence may be higher than the rate for severe husband-to-wife violence in this particular survey due to patterns of underreporting of violence data across gender.

A considerable proportion of the violence that occurs in the United States is associated with alcohol (Leonard and Jacob 1988). A review of the literature on alcohol and violent crime concluded the following: (1) alcohol is involved in at least 50 percent of homicides and assaults; (2) alcohol use prior to a violent episode is high among both assailants and their victims; and (3) homicide victims are more likely than their assailants to have been intoxicated if they provoked the fight (Murdoch et al. 1990). As with other forms of violence, alcohol is thought to play an important role in IPV. An overview of studies of IPV estimated that men were drinking when the violence occurred in about 45 percent of the cases (the range across all studies was 6 to 57 percent) and that women were drinking in about 20 percent of the cases (the range was 10 to 27 percent) (Roizen 1993). Research suggests that compared with other cases, alcohol-related violence is associated with more severe injuries and with more chronic cases of violence (Reider et al. 1988).

Although both men and women perpetrate IPV, violence against women is a greater public health concern for a number of reasons. For example, male-to-female partner violence is more often repeated and is more likely to result in injury and death than violence that is perpetrated by women (Sorenson et al. 1996). In addition, one in three women will be assaulted by an intimate male partner during her lifetime (Browne 1993).

This article discusses some of the theories proposed to explain the differences in rates of IPV among ethnic groups in the United States. The article also reviews and summarizes results from a 1995 national survey on IPV among U.S. couples. The study examined rates of IPV and its relationship with alcohol use among white, black, and Hispanic couples, the three largest ethnic groups in the country.

## Alcohol-Related IPV Varies by Ethnic Group: Proposed Explanations for the Differences

Although IPV occurs among all ethnic groups in the United States, some groups have higher rates than others. For instance, results from one study indicated that the annual rate of severe husband-to-wife violence was twice as high among blacks than among other ethnic minorities and 400 percent greater than the rate for whites (Straus et al. 1980). Another study reported that blacks’ annual rate of severe husband-to-wife violence was 11 percent, compared with 3 percent for whites (Cazenave and Straus 1990) and 7.3 percent for Hispanics (Straus and Smith 1990).

The increased occurrence of IPV among ethnic minority groups compared with whites has been explained in a variety of ways. Two explanations are represented by the “subculture of violence theory” and the “social-structural theory” (see review by Gelles 1985). The subculture of violence theory proposes that certain groups in society accept violence as a means of conflict resolution more than others. Such an acceptance, as the theory title indicates, is considered to be part of the culture of that group. The social-structural theory proposes instead that IPV is associated not with the cultural characteristics of a group but with the social structural conditions (e.g., poverty, undereducation, high unemployment, and racial discrimination) that characterize the lives of members of a particular group.

For instance, several studies have shown that socioeconomic status is an important variable to consider when exploring the association between alcohol use and IPV. Straus and Smith (1990) found that the combined effects of urbanicity of residence, income, and younger age largely explained differences in severe violence between Hispanics and whites. Reanalyzing these same data, Kantor and colleagues (1993) also found that differences in severe husband-to-wife violence among Hispanics and whites were attributable to socioeconomic factors and drinking. These data support the social-structural theory but not the subculture of violence theory (see Gelles 1985). However, differences in severe husband-to-wife violence between blacks and whites cannot be attributed to the effects of socioeconomic factors (Cazenave and Straus 1990) or to the combined effects of socioeconomic factors and alcohol use (Kantor et al. 1993). In other words, when differences in socioeconomic status and alcohol use between blacks and whites are kept constant, blacks’ higher rate of IPV does not disappear.

Other theories explaining the relationship between alcohol use and IPV apply equally to majority and minority groups. The “acute effects hypothesis” states that drinking is associated with IPV because of “alcohol’s power to disinhibit” (Collins and Messerschmidt 1993). A second hypothesis is that alcohol is a convenient factor to excuse behavior that otherwise would be unacceptable. Less well researched but relevant for prevention and intervention is the chronic effects hypothesis (Collins and Messerschmidt 1993). This hypothesis proposes that people with histories of heavy or problem drinking are either predisposed to violence or that such chronic use serves as a marker for other factors related to both violence and alcohol use (e.g., personality and temperament, economic deprivation, and exposure to or experience of abuse as a child). Furthermore, chronic alcohol use might cause or exacerbate organic brain damage associated with aggressive or violent outbursts (Rosenbaum 1989).

## Research Methods Used in the 1995 National Study of Couples

The 1995 National Study of Couples, funded by the National Institute on Alcohol Abuse and Alcoholism, surveyed more than 1,000 white, black, and Hispanic couples concerning episodes of IPV (for more details, see Caetano et al. 2000).

### Survey and Data Collection

The study population was selected with random probability methods to ensure that a diverse group was surveyed. The sample of couples selected is representative of married and cohabiting couples in the 48 contiguous United States, meaning that the results can be applied to all couples in the U.S. population. Black and Hispanic couples were over-sampled, so that the analyses could provide a better description of the characteristics of IPV in these two groups. A total of 1,925 eligible couples age 18 and older were identified in the survey, and 1,635 couples participated for a response rate of 85 percent. Among the couples interviewed, 555 were white, 358 were black, and 527 were of Hispanic origin. The analyses reported here are restricted to these 1,440 couples. This survey obtained reports from both partners, which represents a methodological advancement over previous studies that relied on one person’s report to serve as a proxy for the other partner’s report.

### Ethnic Identification

Respondents who identified themselves as “black of Hispanic origin (i.e., Latino, Mexican, Central or South American, or any other Hispanic origin)” and “white of Hispanic origin (i.e., Latino, Mexican, Central or South American, or any other Hispanic origin)” were classified as Hispanic. Respondents who selected the category “black, not of Hispanic origin” were classified as black. Respondents who selected “white, not of Hispanic origin” were classified as white.

### IPV Definition

Study participants were asked about the occurrence of 11 violent behaviors that they may have perpetrated against their partners or that their partners may have perpetrated against them during the previous year. The violence items were adapted from the Conflict Tactics Scale, Form R (Straus 1990) and included the following: threw something; pushed, grabbed, or shoved; slapped; kicked, bit, or hit; hit or tried to hit with something; beat up; choked; burned or scalded; forced sex; threatened with a knife or gun; used a knife or gun. Separate analyses were conducted for male perpetrated (i.e., male-to-female partner violence [MFPV]) and female perpetrated (i.e., female-to-male partner violence [FMPV]) violence. For both of these variables, violence was considered present when at least one of the partners reported a violent incident. Following an affirmative response, the respondent was then asked if he or she or his or her partner was drinking during the incident. In addition, questions were asked about exposure to violence in childhood and adolescence. Participants reported whether any of the following acts had been perpetrated against them during childhood or adolescence by a parent or other caregiver: hit with something; beaten up; choked, burned, or scalded; threatened with a knife or gun; or had a knife or gun used against them.

### Alcohol Consumption Measures

The respondents were asked to characterize their frequency of drinking over the 12-month period prior to the survey according to 1 of 11 categories ranging from “never” to “three or more times a day.” The survey quantified consumption by asking participants for the proportion of drinking occasions on which they drank 5 or 6, 3 or 4, and/or 1 or 2 glasses each of wine, beer, and liquor. The information on frequency and quantity of alcohol use was combined and used to estimate the volume (i.e., number of drinks) of alcohol consumed in a given time period. A drink was defined as 1 ounce of spirits, a 4-ounce glass of wine, or a 12-ounce can of beer. (See Clark and Hilton 1991 for further details.)

### Alcohol Problem Measures

The survey asked respondents whether they had experienced any problems from a list of 29 alcohol-related problems and to report each of the problems that they had experienced in the 12 months prior to the survey. The items included in the survey reflected 20 years of cumulative experience measuring alcohol-related problems and addressed 14 specific problem areas: salience of drinking, impaired control, withdrawal, relief drinking, tolerance, binge drinking, belligerence, accidents, health-related problems, work-related problems, financial problems, problems with the police, problems with one’s spouse, and problems with other people. For the analysis reported here, respondents were divided into two groups: (1) those who reported at least one problem in the past 12 months and (2) those who reported no problems. The respondents who were defined as having alcohol-related problems for the purposes of this study would not necessarily meet the diagnostic criteria for alcohol abuse or dependence.

## Results

The rates of IPV found in the study are reported here in association with the individual-level factors of ethnicity, drinking during the violent episode, drinking status, and alcohol problems. Environmental factors are discussed in the following section.

### Rates of IPV by Ethnicity

The survey results indicated that 23 percent of black couples reported at least one instance of MFPV. This rate was two times higher than the MFPV rate for white couples (11.5 percent) and 1.3 times higher than the rate for Hispanic couples (17 percent). Rates of FMPV were also higher among black couples (30 percent) than among Hispanic (21 percent) and white couples (15 percent). These cross-ethnic differences in rates of MFPV and FMPV were all statistically significant (Caetano et al. 2000).

Table 1 shows rates of specific types of violence. In general, rates of FMPV events were higher than comparable MFPV events, independent of ethnicity. Statistically significant ethnic differences were found for MFPV events such as pushing, shoving, or grabbing; slapping; kicking, biting, or hitting; hitting with something; and choking. FMPV events with statistically significant differences across ethnic groups included throwing something; pushing, shoving, or grabbing; kicking, biting, or hitting; hitting with something; beating up; forcing sex; and threatening with a gun. It should be noted, however, that because this is a general household sample of couples and not a clinical sample in which severe cases of violence are found, the proportion of couples reporting the most severe types of violence is relatively small.

### Rates of IPV by Drinking During the Violent Episode and Ethnicity

A considerable proportion of the IPV events reported occurred when at least one partner was drinking. Table 2 shows that men were more likely than women to have been drinking during the violent episode. This relationship is independent of ethnicity or whether the violent event being reported was MFPV or FMPV. This difference in rate across genders should be expected, because drinking is more prevalent among men than among women. Across ethnic groups, drinking during the violent event was also more frequent among blacks, independent of gender, than among whites and Hispanics.

### Rates of IPV by Drinking Status and Ethnicity

Another way to look at the relationship between IPV and drinking is to examine the rates of reported IPV among drinkers and abstainers. Table 3 shows that rates of MFPV were much higher among men who reported drinking five or more drinks per occasion at least once per week than among abstainers. This finding is especially true among black men, but differences with abstainers are also large for white and Hispanic men. A minority of the women drinkers reported drinking five or more drinks on an occasion; thus, table 3 also compares rates of FMPV among abstainers and women who drink at least once per week. The greatest differences in rates of FMPV between abstainers and women who drink at least once per week were found among black women. Because abstainers tend to be older and IPV tends to decrease with age, some of the differences between drinkers and abstainers in table 3 could be attributable to age or perhaps to other socioeconomic factors, such as education or income, that were not considered in the analysis.

### Rates of IPV by Alcohol Problems and Ethnicity

People with problems in one area of their lives (e.g., alcohol dependence) are more likely to have problems in other areas of their lives (e.g., IPV) than people without any problems. This clustering of problems in certain people is sometimes referred to as “problem syndrome.” According to this idea, people who have alcohol-related problems (e.g., drinking-and-driving arrests, job problems, tolerance to alcohol, or inability to control drinking) would be more likely to be involved in violent relationships than would people who abstain or drink but do not have alcohol-related problems.

As shown in table 4, couples with alcohol-related problems were more likely than those without problems to report IPV, independent of whether the violence is male- or female-perpetrated. Rates of MFPV were two to four times higher among men with alcohol problems than among men without problems. FMPV was about two times more frequent in relationships where men have alcohol problems than in other relationships. MFPV was more frequent among white and black couples, but not Hispanic couples, in which the woman reported an alcohol problem. FMPV was approximately two times more frequent among couples in which the woman reported alcohol problems compared with other couples. However, these results changed when the analysis controlled for the effects of sociodemographic factors and psychosocial variables, such as impulsivity, relationship length, presence of children in the family, childhood victimization, and approval of aggression. When these factors were considered, alcohol problems among both men and women were associated strongly with IPV among black couples; alcohol-related problems among women, but not among men, were associated with IPV among white couples; and a strong relationship no longer existed between alcohol problems and IPV among Hispanic couples.

## The Role of Neighborhood Characteristics

In addition to examining the relationships between ethnicity, alcohol use, and alcohol problems (i.e., individual-level factors) and IPV, the 1995 National Study of Couples also sought to assess the contribution of environmental factors to the risk of IPV (see Cunradi et al. 2000). Therefore, neighborhood characteristic data from the 1990 Census were added to the original data collected from couples. The types of neighborhood data added included percentage of the population with a high school diploma, percentage of the population age 16 or older who were unemployed, percentage in a blue collar occupation, and neighborhood poverty (i.e., whether 20 percent or more of the census tract’s population had an annual income below the Federal poverty line, which was $12,700 for a family of four). The analyses controlled for individual- and household-level factors, including the average number of drinks consumed per month by each person in the couple and whether either partner reported an alcohol problem. The results indicated that among blacks, couples residing in impoverished neighborhoods were three times more likely than were couples in nonimpoverished neighborhoods to be involved in MFPV. White couples in impoverished neighborhoods were almost four times more likely to report an incident of FMPV than were white couples in other neighborhoods. In addition, Hispanic couples in impoverished neighborhoods were about two times more likely to report FMPV than were other Hispanic couples.

The positive association between residence in an impoverished area and the occurrence of IPV lends support to the social-structural theory of IPV, suggesting that independent of the characteristics of the two people in the couple and of their drinking and alcohol problems, life in a poverty area influences behavior and may lead to IPV. This could possibly occur through a link between poverty, increased stress, increased disharmony between couples, and violence. It is important to try to identify specific characteristics of impoverished neighborhoods that are linked to IPV, which is one goal of the followup to the 1995 National Study of Couples. This study is now under way, and couples interviewed in 1995 are being recontacted for a second wave of interviews. The new questionnaire requests information about a number of neighborhood factors (e.g., social cohesion and crime level) to assess the relationship between these factors and IPV.

## Discussion and Conclusions

This review shows that some important differences exist in rates of and risk factors for IPV across gender and across white, black, and Hispanic couples in the United States. Rates for FMPV are higher than those for MFPV, which is counter-intuitive. Two possible explanations exist for this finding. Males may have underreported violence more than women did, or the association between gender and IPV may differ for a general population sample compared with a clinical sample. Studies with clinical samples consistently show that most of the violence is perpetrated by men.

Most of the differences across ethnic groups were found between white and black couples—blacks reported higher rates of IPV than did whites. The difference between white and black couples remained statistically significant for FMPV, but not for MFPV, when the researchers controlled for factors such as socioeconomic background, drinking, and history of victimization by violence.

Drinking and alcohol-related problems were associated with IPV among white, black, and Hispanic couples. The presence of drinking in a partner-violence incident, however, does not necessarily mean that alcohol is the cause of the violence. It is important to consider that violence often occurs in the absence of alcohol. Therefore, it is possible that the occurrence of violence is not associated with any effect of alcohol, but that alcohol’s role in partner violence may be explained by the expectation that alcohol will have a disinhibitory effect on behavior. It is also possible that some people consciously use alcohol as an excuse for violent behavior and that alcohol appears to be associated with violence, because both heavier drinking and violence have common predictors, such as an impulsive personality.

Other characteristics of individuals besides their drinking and the presence of alcohol-related problems also contribute to the occurrence of IPV. Income level, cohabitation of unmarried partners, unemployment, an impulsive personality, relationship length, violent victimization as a child, observing threats or actual violence between parents, and attitudinal factors (e.g., acceptance of violence) also can act individually or in combination to increase the risk of IPV. Besides these individual-level factors, characteristics of the environment, such as neighborhood poverty, also add to the risk of IPV (Cunradi et al. 2000). Therefore, the combined research evidence suggests that the occurrence of IPV is the result of a constellation of factors that can be summarized as characteristics of the person (e.g., drinking or alcohol problems), of the relationship between partners (e.g., how long they have been together), and of the social environment (e.g., poverty level).

Heavier drinkers are at increased risk for being perpetrators or victims of IPV. People with alcohol problems also seem to be at increased risk for IPV. However, the occurrence of IPV in couples with heavier drinkers or alcohol problems may not necessarily be caused by the drinking or the problems. In some cases (e.g., among whites and Hispanics), controlling for the effect of differences in socioeconomic background, personality variables, and other factors eliminates the association between problems and IPV. In this case, alcohol problems are not the cause of IPV; rather, they are just a marker that is useful in identifying a population which for other reasons has an increased risk of developing violent relationships. Public health professionals interested in prevention and health care professionals delivering alcohol treatment must pay increased attention to these heavier drinkers or problem drinkers in order to minimize the occurrence of IPV. Given the association between alcohol problems and violence reported here, alcohol treatment should help reduce violence among patients to the extent that it also reduces alcohol problems.

In examining the results presented here, we also must take into consideration the sample from which the data were collected. Household samples report mostly “moderate” violence, whereas samples drawn from people in alcohol treatment or shelters for battered women report more severe violence. The findings presented are also limited in some other respects. For example, the study is based on cross-sectional data, and as such, it can only assess associations and not causations. Assessment of causation would require a study with a longitudinal design. Longitudinal research on partner violence is needed to minimize or eliminate temporal ambiguity among some of the variables.

## Figures and Tables

**Table 1:** Prevalence of Intimate Partner Violence by Race/Ethnicity

	Violence Against Intimate Partner							
	
	MFPV (%)				FMPV (%)			
		
Violent Act	White	Black	Hispanic	*x^2^*	White	Black	Hispanic	*x^2^*
Throw something	4.1	5.4	5.8	1.5	9.5	22.1	13.0	11.5**
Push, shove, grab	9.4	19.7	13.0	9.3*	10.2	21.3	13.0	7.4*
Slap	1.5	7.8	5.5	9.3*	4.3	9.7	6.4	4.0
Kick, bite, hit	0.7	4.1	2.6	9.4**	2.7	9.9	5.0	6.9*
Hit with something	0.9	5.1	4.2	15.9***	3.8	15.8	7.5	16.0***
Beat up	0.3	1.4	2.2	4.9	0.0	2.1	1.3	16.5***
Choke	0.4	2.7	1.9	7.4*	0.1	1.4	0.7	4.3
Burn	0.4	0.2	0.0	2.6	0.0	1.2	0.2	3.0
Force sex	0.5	1.7	1.9	5.5	0.4	2.9	1.0	6.6*
Threaten with knife/gun	0.4	0.6	0.7	0.7	0.1	3.1	0.9	9.8**
Use knife/gun	0.3	0.2	0.4	0.3	0.0	0.9	0.5	7.3*

MFPV = male-to-female partner violence; FMPV = female-to-male partner violence; *x^2^* = chi squared. The chi square test is the statistical technique used to determine the probablility value or *p* value.

* *p* < 0.05;

** *p* < 0.01;

*** *p* < 0.001.

NOTE: Rates of female-perpetrated events were generally higher than the rates for comparable male-perpetrated events, independent of ethnicity. It should be noted, however, that because this is a general household sample of couples, and not a clinical sample in which severe cases of violence are found, the proportion of couples reporting the most severe types of violence is relatively small.

SOURCE: Adapted from Caetano et al. 2000.

**Table 2:** Percentage of People Drinking During Partner-Violence Episode by Gender and Ethnicity

	Type of Partner Violence Among Ethnic Groups							
	
	MFPV				FMPV			
		
Gender of Alcohol-Drinking Partner	White	Black	Hisp.	*x^2^*	White	Black	Hisp.	*x^2^*
Male (%)	29	41	29	2.6	27	34	28	1.0
Female (%)	11	24	5	9.3*	15	22	4	14.5**

MFPV = male-to-female partner violence; FMPV = female-to-male partner violence; Hisp. = Hispanic; *x^2^* = chi squared. The chi square test is the statistical technique used to determine the probability value or *p* value (chi-square analysis was conducted across ethnic groups).

* *p* < 0.05;

** *p* < 0.001.

NOTE: Independent of ethnicity, males were more likely than females to have been drinking alcohol during a partner-perpetrated violent episode.

SOURCE: Adapted from Caetano et al. 2000.

**Table 3:** Perpetration of Partner Violence Among Abstainers and Selected Drinkers by Gender and Ethnicity

	Ethnicity of Participating Couples		
	
Drinking Level of Partner-Violence Perpetrator	White (*n* = 555)	Black (*n* = 358)	Hispanic (*n* = 527)
	**Male-to-Female Partner Violence (MFPV) (%)**		
**Male Quantity/Frequency**			
Abstainer	6	18	17
Drinks five or more drinks on occasion at least once per week	19	40	24

	**Female-to-Male Partner Violence (FMPV) (%)**		
**Female Quantity/Frequency**			
Abstainer	12	25	17
Drinks at least once per week	19	57	21

*n* = number of participating couples (including one male partner and one female partner) within each ethnic category indicated.

NOTE: Rates of MFPV were much higher among males who reported drinking five or more drinks per occasion at least once per week than among abstainers. The greatest differences in rates of FMPV between women who abstained and women who drank at least once per week were found among black women.

SOURCE: Adapted from Caetano et al. 2000.

**Table 4:** Correspondence Between Intimate Partner Violence and Alcohol Problems by Gender and Ethnicity

	Ethnicity, Gender, and Alcohol Problems of Those Engaged in Partner Violence					

Partner-Violence Category	White (*n* = 555)		Black (*n* = 358)		Hispanic (*n* = 527)	
Male Alcohol-Related	Alcohol	No Alcohol	Alcohol	No Alcohol	Alcohol	No Alcohol
Problems and Partner	Problems	Problems	Problems	Problems	Problems	Problems
Violence	(*n* = 78)	(*n* = 477)	(*n* = 71)	(*n* = 287)	(*n* = 126)	(*n* = 401)
MFPV (%)	28	9**	56	14**	27	14**
FMPV (%)	31	13**	58	23**	35	17**

Female Alcohol-Related	Alcohol	No Alcohol	Alcohol	No Alcohol	Alcohol	No Alcohol
Problems and Partner	Problems	Problems	Problems	Problems	Problems	Problems
Violence	(*n* = 33)	(*n* = 522)	(*n* = 29)	(*n* = 329)	(*n* = 34)	(*n* = 493)
MFPV (%)	36	10*	61	20**	14	17
FMPV (%)	50	13**	70	27**	37	20

MFPV = male-to-female partner violence; FMPV = female-to-male partner violence; figures in parentheses are numerals (*n*), and all other figures are percentages (percentages are rounded to the nearest tenth).

* *p* < 0.05;

** *p* < 0.01.

NOTE: Couples with alcohol-related problems were more likely than those without problems to report intimate partner violence, independent of whether the violence was male or female perpetrated.

SOURCE: Adapted from Cunradi et al. 2000.
